# Correction: Folate receptor-targeting mesoporous silica-coated gold nanorod nanoparticles for the synergistic photothermal therapy and chemotherapy of rheumatoid arthritis

**DOI:** 10.1039/d5ra90050f

**Published:** 2025-05-14

**Authors:** Xiangyu Li, Yufei Hou, Xiangxue Meng, Ge Li, Fei Xu, Lesheng Teng, Fengying Sun, Youxin Li

**Affiliations:** a School of Life Sciences, Jilin University 2699 Qianjin Street Changchun Jilin 130012 China sunfengying@jlu.edu.cn liyouxin@jlu.edu.cn

## Abstract

Correction for ‘Folate receptor-targeting mesoporous silica-coated gold nanorod nanoparticles for the synergistic photothermal therapy and chemotherapy of rheumatoid arthritis’ by Xiangyu Li *et al.*, *RSC Adv.*, 2021, **11**, 3567–3574, https://doi.org/10.1039/D0RA08689D.

The authors regret that the panel for MTX-GMs in Fig. 6A was shown incorrectly in the original article. The correct version of Fig. 6 is shown here.

**Fig. 6:**
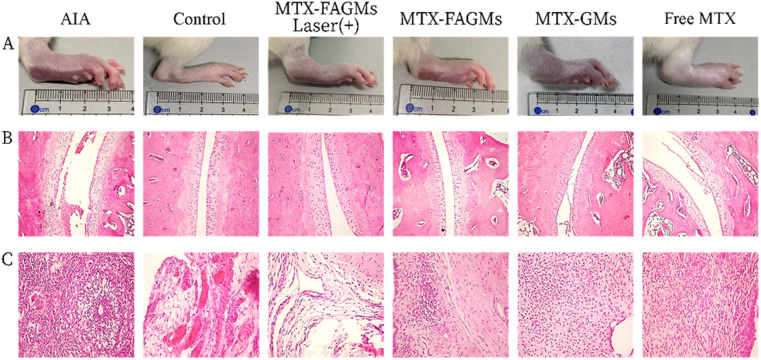
Photographs of the hind paws (A), H&E staining of the ankle joint (B) and periarticular soft tissues (C) of the rats in each group.

An independent expert has viewed the corrected image and has concluded that they are consistent with the discussions and conclusions presented.

The Royal Society of Chemistry apologises for these errors and any consequent inconvenience to authors and readers.

